# Prevalence and associated factors of frailty in patients with chronic kidney disease: a cross-sectional analysis of PEAKING study

**DOI:** 10.1007/s11255-023-03720-z

**Published:** 2023-08-09

**Authors:** Changyuan Yang, Cuixia Xiao, Jiahao Zeng, Ruolan Duan, Xitao Ling, Jiamei Qiu, Qin Li, Xindong Qin, La Zhang, Jiasheng Huang, Jiawei He, Yifan Wu, Xusheng Liu, Haijing Hou, Bengt Lindholm, Fuhua Lu, Guobin Su

**Affiliations:** 1 The Second Clinical Medical College, Guangzhou University of Chinese Medicine, Guangzhou City, 510000 China; 2 Department of Nephrology, Guangdong Provincial Hospital of Chinese Medicine, the Second Affiliated Hospital, GuangzhouUniversity of Chinese Medicine, Guangzhou, 510000 China; 3 Department of Nephrology, Shenzhen Hospital, Guangzhou University of Chinese Medicine, Shenzhen City, 518000 China; 4 Department of Nephrology, Peking University First Hospital, Beijing City, 100034 China; 5 Division of Renal Medicine and Baxter Novum, Department of Clinical Science, Intervention and Technology, Karolinska Institutet, 11228 Stockholm, Sweden; 6 Department of Medical Epidemiology and Biostatistics, Karolinska Institute, 11228 Stockholm, Sweden

**Keywords:** Chronic kidney disease, Frailty, Prevalence, Associated factors, Cross-sectional study

## Abstract

**Aim:**

Frailty is common and is reported to be associated with adverse outcomes in patients with chronic diseases in Western countries. However, the prevalence of frailty remains unclear in individuals with chronic kidney disease (CKD) in China. We examined the prevalence of frailty and factors associated with frailty in patients with CKD.

**Methods:**

This was a cross-sectional analysis of 177 adult patients (mean age 54 ± 15 years, 52% men) with CKD from the open cohort entitled Physical Evaluation and Adverse outcomes for patients with chronic Kidney disease IN Guangdong (PEAKING). Frailty at baseline were assessed by FRAIL scale which included five items: fatigue, resistance, ambulation, illnesses, and loss of weight. Potential risk factors of frailty including age, sex, body mass index, and daily step counts recorded by ActiGraph GT3X + accelerometer were analyzed by multivariate logistic regression analysis.

**Results:**

The prevalence of prefrailty and frailty was 50.0% and 11.9% in patients with stages 4–5 CKD, 29.6% and 9.3% in stage 3, and 32.1% and 0 in stages 1–2. In the multivariate logistic regression analysis, an increase of 100 steps per day (OR = 0.95, 95% CI 0.91–0.99, *P* = 0.01) and an increase of 5 units eGFR (OR = 0.82, 95% CI 0.68–0.99, *P* = 0.045) were inversely associated with being frail; higher BMI was associated with a higher likelihood of being frail (OR = 1.52, 95% CI 1.11–2.06, *P* = 0.008) and prefrail (OR = 1.25, 95% CI 1.10–1.42, *P* = 0.001).

**Conclusion:**

Frailty and prefrailty were common in patients with advanced CKD. A lower number of steps per day, lower eGFR, and a higher BMI were associated with frailty in this population.

**Supplementary Information:**

The online version contains supplementary material available at 10.1007/s11255-023-03720-z.

## Introduction

Chronic kidney disease (CKD), characterized by progressive loss of kidney function, represents a global public health problem due to its high prevalence and the heavy medical burden it imposes, causing more than 1.2 million deaths and 28 million years of life loss per year [1, 2].

Frailty, a clinical syndrome characterized by the decrease of physiological reserves and the increase of external dependence and disease susceptibility [3] is more common in patients with CKD [4] than that in non-CKD populations [5, 6]. Frailty has been reported to be associated with adverse outcomes including mortality [7–9], hospitalization [9–12], decreased renal function [13], and dialysis-related complications [14] in patients with CKD. The reported prevalence of frailty in patients with CKD varies, ranging from 7 to 73% in different populations [4]. This wide range of frailty prevalence can in part be attributed to regional differences, and differences in the severity of the disease or the tools of frailty assessment. Previous studies were performed predominantly in European and American populations [4] and in most cases in dialysis patients [14]. Little is known about the prevalence of frailty in Chinese patients with non-dialysis CKD (ND-CKD). Among several tools to evaluate the degree of frailty, the FRAIL scale (FS) has emerged as a valid and efficient tool in clinical practice [15–17]. The evaluation process takes less than 5 min, making it one of the most convenient tools in crowded clinical settings. However, the prevalence of frailty assessed by FS is still uncertain in Chinese patients with ND-CKD.

Considering the adverse outcomes associated with frailty, it is of great clinical and economic significance to identify individuals with high risk of frailty early in patients with CKD and to understand the associated factors of frailty in this population. Thus, this study aimed to examine the prevalence of prefrailty and frailty as defined by FS and their associated factors  in Chinese patients with ND-CKD.

## Methods

### Study design

This is a cross-sectional analysis of adult patients from the Physical Evaluation and Adverse outcomes for patients with chronic Kidney disease IN Guangdong, China (PEAKING) study. The results of this study are reported according to STROBE (Strengthening the Reporting of Observational Studies in Epidemiology) guidelines [18].

### PEAKING cohort and setting

The PEAKING study is an open cohort study established in 2017 and aims to investigate the level of physical activity and adverse outcomes in patients with CKD. Patients registered in the chronic disease management clinic of Guangdong Provincial Hospital of Chinese Medicine (A tertiary hospital of the region, located in Guangzhou city, the capital city of Guangdong province, China) were invited if they met the following inclusion criteria: (1) age over 18 years; (2) diagnosed as CKD with estimated glomerular filtration rate (eGFR) less than 60 ml/min/1.73 m^2^ or abnormal kidney biomarkers (such as proteinuria, hematuria, etc.) for more than 90 days [19]. Patients were not invited if they met the following exclusion criteria: (1) has received or expected to receive kidney replacement therapy within 1 year; (2) pregnant or lactating women or those planning pregnancy within 1 year; (3) acute myocardial infarction or acute cerebrovascular event, acute obstructive nephropathy requiring surgery within 3 months; (4) severe arrhythmia or heart failure (New York Heart Association class grade III or above) which could not be controlled by medication; (5) active malignant tumor, decompensated cirrhosis or diseases of the hematopoietic system; (6) serious mental illness, or unable to cooperate with the investigation and treatment due to other reasons; (7) physically disability to perform physical activities;

In our study, participants were included in the analysis if they met the following: (1) complete baseline demographics and laboratory test data; (2) valid accelerometer data. The study was approved by the Ethics Committee of Guangdong Provincial Hospital of Chinese Medicine (B2015-152-02), and all participants gave informed consent.

### Assessment of frailty

FRAIL scale (FS) was used to evaluate the degree of frailty at baseline as recommended by the International Conference of Frailty and Sarcopenia Research (ICFSR) [20]. FS has been used in patients with all stages of CKD and demonstrated good reliability and validity [14, 21]. FS is a self-report tool and derives its name from the five domains: fatigue, resistance, ambulation, illness, and loss of weight [22]. FS score ranges from zero to 5 points. One domain represents 1 point. Those with a score more than 2 points are deemed to be with frail, 1 to 2 points are considered as prefrail stage, and those with zero points are classified as robust (Supplement Table 1).

### Measurements of potential risk factors of frailty

Upon enrollment, a case report form (CRF) was used to collect baseline data of participants, including demographic data (sex, age, marital status, educational level, health insurance, working status, smoking and alcohol drinking habit). Comorbidities such as hypertension, diabetes, cardiovascular disease, stroke, chronic obstructive pulmonary disease, bronchial asthma, arthritis, malignant tumor, and osteoporosis were recorded on the CRF allowing calculation of Charlson comorbidity index (CCI) [23]. Information about the kidney disease was collected as well, including primary disease of CKD, disease duration, and kidney biopsy report if available.

Participants underwent physical examinations at baseline for anthropometric parameters including body weight and height, and waist circumference. Body mass index (BMI) was calculated accordingly and categorized as severely underweight (< 16.5 kg/m^2^), underweight (< 18.5 kg/m^2^), normal (18.5–22.9 kg/m^2^), and overweight (> 23 kg/m^2^) [24]. In addition, their physical performance and physical activity were measured using established approaches: handgrip strength was measured as a proxy for upper limb performance using a digital handgrip dynamometer (EH101, CAMRY Sensun Weighing Apparatus Group Ltd, Guangdong, China), calculated from the largest readings of three measurements using participants’ dominant hand; the ActiGraph GT3X + accelerometers (ActiGraph, LLC Pensacola, FL, USA) were employed to evaluate daily step counts as a reliable and objective measure of physical activity. It incorporates a triaxial accelerometer that detects and records acceleration forces in different directions. These forces are then converted into activity counts, which reflect the intensity and duration of movement. This sophisticated device has gained widespread adoption by over 1500 colleges and institutions in more than 65 countries or regions worldwide [25]. They serve as precise instruments for capturing and assessing multiple facets of physical activity, encompassing not only daily step counts but also capturing the nuances of intensity, duration, and frequency of physical activity, sedentary behavior, and energy expenditure. Subjects were required to wear an accelerometer on the right hip in daytime for 9 consecutive days [26]. Data were considered valid if a participant had at least 3 days (including one non-working day) of at least 10 hours per day recorded [27]. We selected an epoch length of 60 seconds [28]. A non-wear-time was defined as an interval of at least 60 min of zero activity counts [28]. At the end of the measurement period, the accelerometer data were retrieved through the ActiLife software. Daily step counts were defined as the average of daily step counts from valid wearing days.

Finally, participants were required to have laboratory tests at baseline including the number of white blood cell, percentage of neutrophils, hemoglobin, estimated glomerular filtration rate (eGFR) estimated by CKD-EPI Creatinine Eq. (2021) [29], spot urine protein–creatinine ratio, serum potassium, total serum calcium, serum albumin, serum uric acid, serum triglyceride, and serum total cholesterol.

### Statistical analysis

Data were described as percentages (%) for categorical variables and mean and standard deviation (M ± SD) or median and interquartile range [M(IQR)] for continuous variables. To test the statistical difference among groups (robust, prefrail and frail), univariate analyses were conducted using Chi-squared test for categorical variables and analysis of variance (with post hoc Tukey analysis) or Kruskal–Wallis test (with Mann–Whitney *U* test for post hoc analysis) for continuous variables as appropriate. Variables that showed statistical significance of *P* < 0.1 in the univariate analyses were included in the multivariate logistic regression analysis, which estimated the odds ratio (OR) and 95% confidence interval (CI) with the robust group as reference group. We further treated FS score as a continuous dependent variable and examined associated factors of FS score in multiple linear regression analysis with stepwise backward variable selection, incorporating the same panels of variables in the aforementioned logistic regression. A two-tailed examination with *P* value less than 0.05 was used as an indication of statistical significance. All analyses were conducted using the Stata 15.0 and the free statistics analysis platform.

## Results

**Fig. 1:**
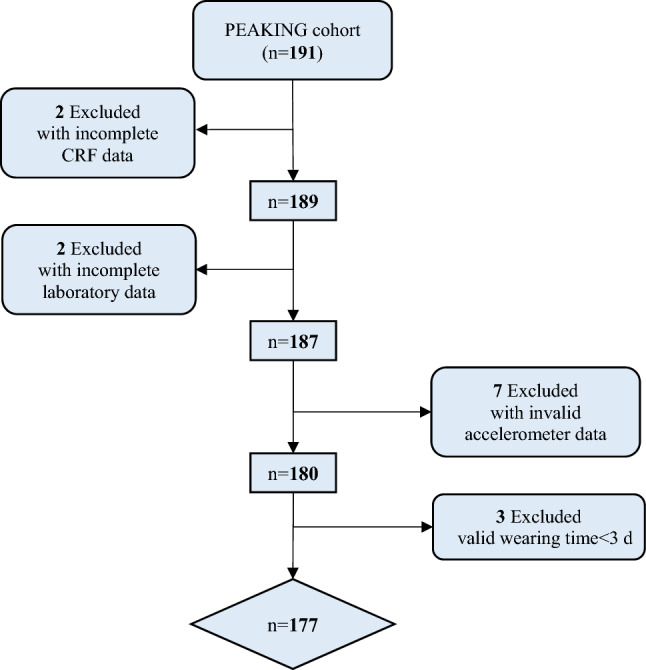
Flow chart of patient selection. *CRF* case report form, *PEAKING* Physical Evaluation and Adverse outcomes for patients with chronic Kidney disease IN Guangdong

A total of 191 eligible ND-CKD adults were enrolled from the PEAKING cohort. After excluding patients with incomplete CRF data (*n* = 2), incomplete laboratory data (*n* = 2), and invalid accelerometer data (*n* = 10), 177 patients were included in this cross-sectional study (Fig. 1).

The mean age of participants was 54 ± 15 years and 92 (52%) were men. There were 42 (23.7%) participants with CKD stages 4–5, 54 (30.5%) with CKD stages 3a–3b, and 81 (45.8%) had CKD stages 1–2. The majority of the participants had high school or higher education (78.6%), were married (88.7%), had medical insurance (79.1%), were non-smokers (88.7%) and non-alcohol drinkers (98.3%) (Table 1). According to FRAIL assessment, 104 (58.8%) were robust, 63 (35.6%) were prefrail, and 10 (5.6%) were frail (Table 1). The prevalence of prefrailty and frailty was 50.0% and 11.9% in patients with stages 4–5 CKD, 29.6% and 9.3% in stages 3a-3b, and 32.1% and 0 in stages 1–2. CKD patients with frailty were more likely to be older (*P* < 0.001).

**Table 1:** Demographic characteristics of Chinese patients with non-dialysis chronic kidney disease by different degree of frailty

	Robust (*n* = 104)	Prefrail (*n* = 63)	Frail (*n* = 10)	*P* value
Age (years)^a^	51.9 ± 14.8	54.1 ± 13.4	70.8 ± 8.8	** < 0.001**
Male, No. (%)^b^	57 (54.8)	30 (47.6)	5 (50.0)	0.678
Education level, No. (%)^b^				0.112
Primary school	6 (5.8)	2 (3.2)	3 (30.0)	
Middle school	14 (13.5)	12 (19.0)	1 (10.0)	
High school	29 (27.9)	22 (34.9)	3 (30.0)	
College or higher	55 (52.9)	27 (42.9)	3 (30.0)	
Married, No. (%)^b^	88 (84.6)	57 (90.5)	9 (90.0)	0.532
Employed, No. (%)^b^	60 (57.7)	31 (49.2)	4 (40.0)	0.385
Having health insurance, No. (%)^b^	82 (78.8)	49 (77.8)	9 (90.0)	0.863
Ex-/current-smoker, No. (%)^b^	22 (21.2)	18 (28.6)	5 (50.0)	0.102
Ex-/current alcohol drinker, No. (%)^b^	12 (11.5)	8 (12.7)	1 (10.0)	0.926

The *P* values indicated in bold are statistically significant (*P* < .05)

^a^Continuous variables are shown as mean and standard deviation and were analyzed using analysis of variance

^b^Categorical variables are shown as number of cases (percentage) and were analyzed using the Chi-squared test

The mean BMI of participants was 22.2 ± 3.0 kg/m^2^, which increased as the frail degree increased (up to 90% of participants were within the normal range or overweight). The mean daily step counts of 7532 ± 3268 gradually decreased as the degree of frailty increased. As expected, frail patients were more likely having a lower level of handgrip strength (*P* = 0.008), less daily step counts (*P* < 0.001), lower eGFR (*P* = 0.01), and higher CCI scores (*P* < 0.001). Prefrail participants were more likely to have a higher CCI score (*P* = 0.014), higher BMI (*P* = 0.022), and lower eGFR (*P* = 0.31) (Table 2).

**Table 2:** Clinical characteristics and laboratory test of Chinese patients with non-dialysis chronic kidney disease by different degree of frailty

	Robust (*n* = 104)	Prefrail (*n* = 63)	Frail (*n* = 10)	*P* value
Duration of disease (year)^a^	4 (2, 7)	6 (2, 12)	4.5 (2, 18)	0.486
Causes of CKD, No. (%)^b^				0.154
IgA nephropathy	19 (18.3)	12 (19.0)	0	
Other primary glomerulonephritis	38 (36.5)	16 (25.4)	1 (10.0)	
Diabetic kidney disease	5 (4.8)	7 (11.1)	2 (20.0)	
Benign renal arteriosclerosis	7 (6.7)	4 (6.3)	1 (10.0)	
Unknown reasons	35 (33.7)	24 (38.1)	6 (60.0)	
Comorbidities, No. (%)^b^				
Hypertension	49 (47.1)	37 (58.7)	7 (70.0)	0.187
Diabetes mellitus	10 (9.6)	11 (17.5)	4 (40.0)	**0.024**
Cardiovascular disease	6 (5.8)	5 (7.9)	3 (30.0)	0.056
Cerebrovascular accident	2 (1.9)	4 (6.3)	1 (10.0)	0.140
Chronic pulmonary disease	1 (1.0)	1 (1.6)	0	1.000
Autoimmune rheumatic diseases	6 (5.8)	1 (1.6)	1 (10.0)	0.180
Gastrointestinal diseases	22 (21.2)	11 (17.5)	2 (20.0)	0.861
LGPSD	18 (17.3)	7 (11.1)	2 (20.0)	0.505
Tumor	6 (5.8)	4 (6.3)	2 (20.0)	0.208
Musculoskeletal diseases	11 (10.6)	6 (9.5)	1 (10.0)	1.000
CCI^a^	0 (0, 2)	2 (0, 3)	4 (2, 6.25)	** < 0.001**
Physical parameters				
Body height (m)^c^	1.63 ± 0.07	1.62 ± 0.08	1.59 ± 0.13	0.246
Body weight (kg)^a^	58.10 (51.75, 65.00)	60.00 (53.50, 67.80)	54.40 (49.28, 67.95)	0.486
Body mass index (kg/m^2^)^c^	21.72 ± 2.84	22.98 ± 3.21	23.10 ± 2.57	**0.020**
Waist circumference (cm)^c^	80.28 ± 9.66	83.04 ± 10.25	84.00 ± 13.06	0.167
Physical performance and activity				
Handgrip strength (kg)^c^	31.37 ± 8.15	29.82 ± 9.40	22.78 ± 6.03	**0.009**
Daily step counts (×100)^c^	80.68 ± 31.61	72.63 ± 32.27	36.67 ± 15.40	** < 0.001**
eGFR (mL/min/1.73m^2^)^a^	63.5 (39, 88)	55 (22, 76)	30.5 (15, 49)	**0.002**
Spot urine protein–creatinine ratio (g/g)^a^	0.65 (0.12, 0.90)	0.57 (0.19, 1.63)	0.20 (0.12, 0.66)	0.110
White blood cell counts (× 10^9^/L)^c^	6.45 ± 1.54	6.58 ± 1.90	7.23 ± 1.74	0.374
Neutrophils (%)^c^	58.73 ± 8.78	60.67 ± 8.85	63.82 ± 5.19	0.116
Hemoglobin (g/L)^c^	129.14 ± 18.93	123.68 ± 20.55	119.20 ± 16.79	0.101
Serum albumin (g/L)^c^	44.18 ± 3.76	45.50 ± 3.78	44.76 ± 4.76	0.088
Serum uric acid (umol/L)^c^	391.17 ± 76.12	395.45 ± 95.87	420.60 ± 110.86	0.580
Serum triglyceride (mmol/L)^c^	1.41 ± 0.54	1.58 ± 0.59	1.63 ± 0.57	0.129
Serum total cholesterol (mmol/L)^c^	4.84 ± 0.86	4.81 ± 0.96	4.57 ± 0.46	0.645
Serum potassium (mmol/L)^c^	4.39 ± 0.42	4.49 ± 0.53	4.50 ± 0.56	0.334
Serum total calcium (mmol/L)^a^	2.36 (2.32, 2.42)	2.34 (2.28, 2.40)	2.36 (2.30, 2.44)	0.101

The *P* values indicated in bold are statistically significant (*P* < .05)

*CKD* chronic kidney disease, *LGPSD* liver, gallbladder, pancreas, and spleen diseases, *CCI* Charlson comorbidity index, *eGFR* estimated glomerular filtration rate calculated by CKD-EPI Creatinine Eq. (2021)

^a^Continuous variables are shown as median and interquartile rang and were analyzed using Kruskal–Wallis test

^b^Categorical variables are shown as number of cases (percentage) and were analyzed using the Chi-squared test or Fisher test

^c^Continuous variables are shown as mean and standard deviation and were analyzed using analysis of variance

After univariate analyses, six potential indicators (*P* < 0.1) associated with frailty or prefrailty in ND-CKD patients, including age, BMI, handgrip strength, daily step counts, eGFR, and serum albumin, were included in the multivariate logistic regression analysis. Because the frailty assessment tool adopted in this study includes the module of comorbidity, comorbidity-related factors (diabetes mellitus, cardiovascular disease, and CCI) are excluded in the multivariate logistic regression analysis.

### Factors associated with prefrailty in patients with ND-CKD in multivariate logistic regression

**Fig. 2:**
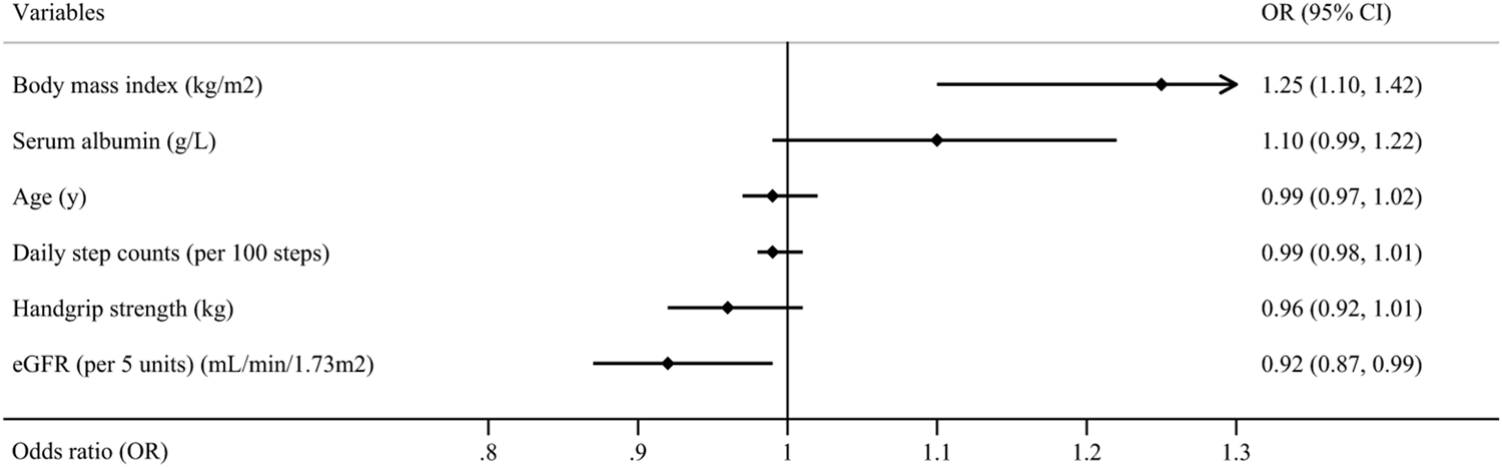
Factors associated with prefrailty in Chinese patients with non-dialysis chronic kidney disease in multivariate logistic regression analysis. eGFR: estimated glomerular filtration rate

Higher BMI (OR = 1.25, 95% CI 1.10–1.42, *P* = 0.001) was associated with a higher likelihood of being prefrail; an increase of 5 units eGFR (OR = 0.92, 95% CI 0.87–0.99, *P* = 0.008) was inversely associated with being prefrail (Fig. 2).

### Factors associated with frailty in patients with ND-CKD in multivariate logistic regression

**Fig. 3:**
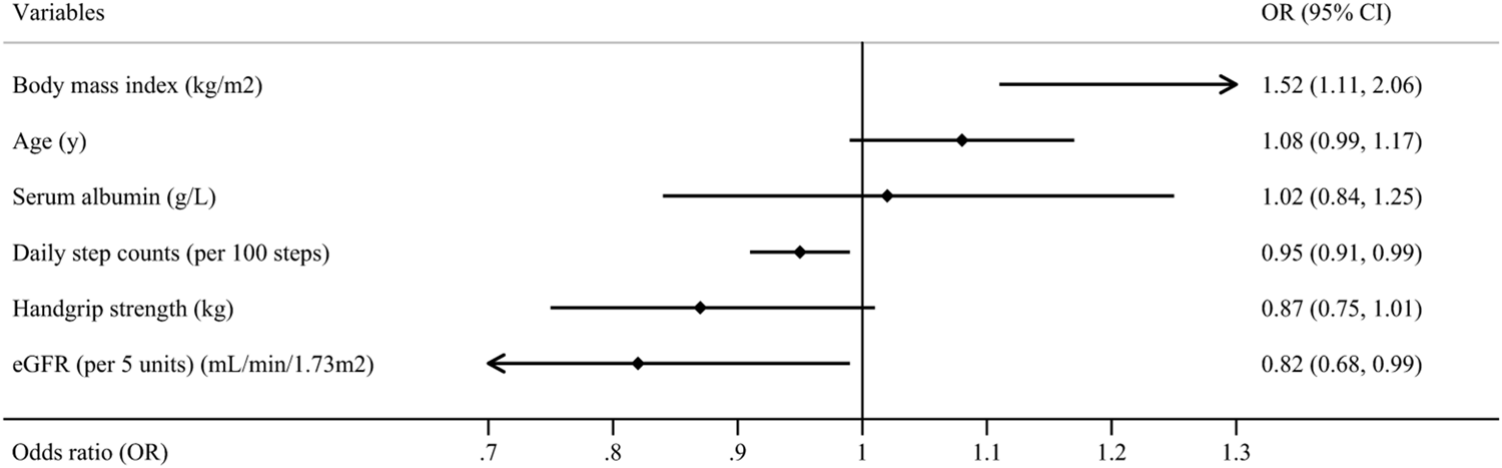
Factors associated with frailty in Chinese patients with non-dialysis chronic kidney disease in multivariate logistic regression analysis. eGFR: estimated glomerular filtration rate

An increase of 100 steps per day (OR = 0.95, 95% CI 0.91–0.99, *P* = 0.01) and an increase of 5 units eGFR (OR = 0.82, 95% CI 0.68–0.99, *P* = 0.045) were inversely associated with being frail, and higher BMI was associated with a higher likelihood of being frail (OR = 1.52, 95% CI 1.11–2.06, *P* = 0.008) (Fig. 3).

In the multiple linear regression analysis, with FS scores as the dependent variable, daily step counts (*β* = − 0.008, *P* < 0.001) and eGFR (β = − 0.005, *P* < 0.013) were associated with a lower severity of frailty, BMI (*β* = 0.074, *P* = 0.001) was associated with a higher severity of frailty among patients with ND-CKD.

## Discussion

In this cross-sectional analysis of the PEAKING study on physical status and physical activity versus outcomes in patients with CKD, people with ND-CKD, especially in those with advanced CKD, prefrailty and frailty were common. Daily step counts, BMI, and eGFR were independent associated factors for frailty in this population. Our study highlights the frailty burden in patients with ND-CKD and indicates that daily steps and BMI may be used as a marker of frailty in this population.

Prefrailty was quite common (41.2%) among patients with ND-CKD while the prevalence of frailty was only 5.6%, which is slightly lower than that reported in a systematic review where the prevalence of frailty ranged from 7.0 to 42.6% among patients with ND-CKD [4]. This may be attributed to the healthier population included in this study who were all outpatient patients and most of them had relatively high eGFR. It has been reported that the prevalence of frailty increased as the eGFR decreased [13]. Therefore, we further estimated the prevalence of frailty stratified by stage of CKD and found that the prevalence of frailty was 11.9% in stages 4–5 CKD and 9.3% in stages 3a–3b CKD, which is consistent with previous study [30].

In our study, we found even small increase in daily step counts, such as 100 steps, was inversely associated with frailty in CKD patients. There are several potential explanations. On the one hand, step counts are the most common indicator of physical activity. It incorporates both light and moderate-to-vigorous physical activity and has been become a common method of assessing daily physical activity regardless of location, culture, age, and gender [31]. Increasing step counts in daily life is considered as one of the most reasonable and cost-effective approaches for reducing the risk of several diseases including frailty [32, 33]. For example, Daiki et al. found that increasing the current step count by as little as 1,000 steps/day (about 10 min of activity) may potentially prevent frailty in older adults [33]. On the other hand, the number of daily steps may correlate to skeletal muscle mass and it has been reported that frail elderly can increase skeletal muscle mass maintenance by increasing the daily step counts [34], especially in those with lower daily step counts [35]. Our finding lends support to the possibility to extend the evidence from older adults to patients with ND-CKD. Admittedly, we acknowledged that daily step counts might overlap with the ambulation domain of the FRAIL scale to some degree. However, it should be noted that the ambulation domain primarily emphasizes the dependence of frail patients on external support during walking. It does not quantify the extent of ambulation contributes to the status of frailty.

Another important finding of our study was that BMI was independently associated with a higher likelihood of both being frail and prefrail, which are in line with previously published results [10, 13]. There are several potential explanations. First, obesity, as defined by a high BMI, has been associated with decreased physical function and increased fatigue, which are key components of frailty. This decrease in physical function may be a result of the additional stress placed on the body by overweight, leading to physical inactivity and decreased muscle mass and strength [36]. Second, higher BMI is often associated with more adipose tissue, which can promote the development of inflammation, metabolism and transmission of metabolic information between different organs by secreting cytokines such as adiponectin, interleukin-6, and tumor necrosis factor, and ultimately lead to frailty [37].

On the other hand, it should be noted that most of the participants in current study had moderate or higher BMI and no one had extremely low BMI. Previous study has also found extremely low BMI was associated with higher risk of frailty in patients with CKD due to malnutrition, decreased muscle mass, and decreased physical function [38]. Therefore, it may be worthwhile to note that interventions should be directed toward moderate or higher BMI, but not extremely low, in patients with CKD.

The current study also found that eGFR was inversely associated with frailty and prefrailty. This relationship may partially be explained by the increased prevalence of associated comorbidities in patients with CKD, including cardiovascular disease, anemia, and mineral and bone disorders, all of which have been shown to contribute to an increased risk of frailty [1]. Additionally, a decline in kidney function, as indicated by a declining eGFR, is also associated with decreased physical function, another key component of frailty [39].

Our study has some strengths. We have evaluated the current prevalence of frailty and prefrailty in a representative sample of patients with ND-CKD in China. A wide range of clinical and physical parameters were investigated as potential predictors of frailty and prefrailty in this population. The use of ActiGraph GT3X+, one of the most accurate assessment tools in the field of physical activity, to record daily steps strongly enhances the validity of our approach. Nonetheless, several limitations still warrant attention. First, this study is cross-sectional in nature, and we may not be able to confirm the causal relationship between associated factors and frailty. Second, we might underestimate the prevalence of frailty since we excluded patients with physical disability. Third, data on other potential predictors of frailty such as mental health were not available in our study and prevented us from further investigating the relationship between these factors and frailty. Future studies are needed to explore the predictors of frailty among adults with CKD using a prospective longitudinal design with a larger sample size in other settings.

In conclusion, frailty and prefrailty were common in patients with ND-CKD. Daily step counts, BMI, and eGFR were associated with frailty in this population. The findings of this study may provide direction for future longitudinal studies to determine the causal relationship between these factors and frailty in patients with CKD, thereby implementing targeted potential interventions to address the risk of frailty in this population.

### Supplementary Information

Below is the link to the electronic supplementary material.

Supplementary file1 (DOCX 19 kb)

## Declarations

### Data availability

The data that support the findings of this study are available from PEAKING study upon reasonable request. The datasets used and/or analyzed during the current study are also available in the Guangdong Provincial Hospital of Chinese Medicine.
