# Supplementary material for: Prevalence and associated factors of frailty in patients with chronic kidney disease: a cross-sectional analysis of PEAKING study
Source: Int Urol Nephrol. 2023 Aug 9;56(2):751–8. doi: 10.1007/s11255-023-03720-z (PMC10808408; doi:10.1007/s11255-023-03720-z)
Supplement: Supplementary file 1 — Supplementary file1 (DOCX 19 kb) [file 11255_2023_3720_MOESM1_ESM.docx]

**Supplement Table 1**

**FRAIL scale**

| **Num** | **Domains** | **Questions** | **Score** |
| --- | --- | --- | --- |
| 1 | Fatigue | How much of the time during the past 4 weeks did you feel tired? | 1 = All of the time, 2 = Most of the time,  3 = Some of the time, 4 = A little of the time, 5 = None of the time.  Responses of “1” or “2” are scored as 1 and all others as 0. |
| 2 | Resistance | By yourself and not using aids, do you have any difficulty walking up 10 stairs without resting? | 1 = Yes, 0= No |
| 3 | Ambulation | By yourself and not using aids, do you have any difficulty walking several hundred yards? | 1 = Yes, 0 =No |
| 4 | Illness | Did a doctor ever tell you that you have [illness]? The illnesses include hypertension, diabetes, heart attack, stroke, chronic lung disease, congestive heart failure, kidney disease, cancer (other than a minor skin cancer), angina, asthma, and arthritis. | 1 = Yes, 0 = No.  The total illnesses (0–11) are recoded as 0–4 = 0, and 5–11 = 1 |
| 5 | Loss of weight | How much do you weigh with your clothes on but without shoes? [current weight]; One year ago in (MO, YR), how much did you weigh without your shoes and with your clothes on? [weight 1 year ago] | Percent weight change is computed as: [[weight 1 year ago - current weight]/weight 1 year ago]] * 100  Percent change > 5 (representing a 5% loss of weight) is scored as 1, and < 5 as 0 |
